# Disruption of cerebellar microzonal organization in GluD2 (GluRδ2) knockout mouse

**DOI:** 10.3389/fncir.2013.00130

**Published:** 2013-08-20

**Authors:** Miki Hashizume, Taisuke Miyazaki, Kenji Sakimura, Masahiko Watanabe, Kazuo Kitamura, Masanobu Kano

**Affiliations:** ^1^Department of Neurophysiology, Graduate School of Medicine, The University of TokyoTokyo, Japan; ^2^Department of Anatomy, Graduate School of Medicine, Hokkaido UniversitySapporo, Japan; ^3^Department of Cellular Neurobiology, Brain Research Institute, Niigata UniversityNiigata, Japan; ^4^CREST, Japan Science and Technology AgencyKawaguchi, Japan; ^5^PRESTO, Japan Science and Technology AgencyKawaguchi, Japan

**Keywords:** cerebellum, inferior olive, Purkinje cell, climbing fiber, olivo-cerebellar loop, microzone, complex spike

## Abstract

Cerebellar cortex has an elaborate rostrocaudal organization comprised of numerous microzones. Purkinje cells (PCs) in the same microzone show synchronous activity of complex spikes (CSs) evoked by excitatory inputs from climbing fibers (CFs) that arise from neurons in the inferior olive (IO). The synchronous CS activity is considered to depend on electrical coupling among IO neurons and anatomical organization of the olivo-cerebellar projection. To determine how the CF–PC wiring contributes to the formation of microzone, we examined the synchronous CS activities between neighboring PCs in the glutamate receptor δ2 knockout (GluD2 KO) mouse in which exuberant surplus CFs make ectopic innervations onto distal dendrites of PCs. We performed *in vivo* two-photon calcium imaging for PC populations to detect CF inputs. Neighboring PCs in GluD2 KO mice showed higher synchrony of calcium transients than those in wild-type (control) mice. Moreover, the synchrony in GluD2 KO mice hardly declined with mediolateral separation between PCs up to ~200 μm, which was in marked contrast to the falloff of the synchrony in control mice. The enhanced synchrony was only partially affected by the blockade of gap junctional coupling. On the other hand, transverse CF collaterals in GluD2 KO mice extended beyond the border of microzone and formed locally clustered ectopic synapses onto dendrites of neighboring PCs. Furthermore, PCs in GluD2 KO mice exhibited clustered firing (Cf), the characteristic CF response that was not found in PCs of wild-type mice. Importantly, Cf was often associated with localized calcium transients in distal dendrites of PCs, which are likely to contribute to the enhanced synchrony of calcium signals in GluD2 KO mice. Thus, our results indicate that CF signals in GluD2 KO mice propagate across multiple microzones, and that proper formation of longitudinal olivo-cerebellar projection is essential for the spatiotemporal organization of CS activity in the cerebellum.

## Introduction

The cerebellum consists of several parasagittal zonal compartments elongated along rostrocaudal direction (designated A, B, C1–3, and D1–2). These compartments are based on the topography of the olivo-cerebellar projection system that constitutes one of the two major afferent systems to the cerebellum (Groenewegen et al., 1979; Voogd and Glickstein, 1998; Sugihara et al., 2001; Sugihara, 2005). These cerebellar zones are thought to be involved in the control of different aspects of posture, movement and motor coordination (Buisseret-Delmas and Angaut, 1993; Horn et al., 2010). Moreover, several physiological studies have revealed that each cerebellar zone is composed of smaller functional units, called microzones (Andersson and Oscarsson, 1978; Lang et al., 1999; Lang, 2002; Apps and Garwicz, 2005). Detailed morphological studies have revealed that axons of neurons in the inferior olive (IO) branch into about 7 climbing fibers (CFs) along rostrocaudal axis of the cerebellum, and each CF innervates a single Purkinje cell (PC) (Sugihara et al., 1999, 2001; Sugihara and Shinoda, 2004). Experiments with a small injection of anterograde tracer into the IO have demonstrated that a small number of adjacent IO neurons project CFs within narrow longitudinal bands of about 200 μm width (Sugihara et al., 2001; Sugihara and Shinoda, 2004), which correspond to physiologically identified microzones (Lang et al., 1999). Because of this anatomical organization and electrical coupling among adjacent IO neurons, CF inputs are synchronized among PCs within a microzone, and the synchrony rapidly falls off as the mediolateral separation between PCs increases (Llinas and Yarom, 1981; Sotelo et al., 1986; Blenkinsop and Lang, 2006; Ozden et al., 2009; Schultz et al., 2009).

A major hypothesis as to the function of the olivo-cerebellar system is that CFs convey error signals to PCs between the intention and the result of movement (Ito, 2011). Defects in the olivo-cerebellar system results in impairment of motor control and coordination (Chen et al., 2010; Horn et al., 2010; Ito, 2011). It has been shown that several mutant mice that are impaired in developmental CF synapse elimination exhibit ataxia (Chen et al., 1995; Kano et al., 1995, 1998; Kashiwabuchi et al., 1995; Offermanns et al., 1997; Hirai et al., 2005b), suggesting that proper wiring of CFs to PCs is important for motor coordination. Among these examples, the mutant mouse deficient in ionotropic glutamate receptor δ2 subtype (GluD2) is best characterized by morphological, electrophysiological, and behavioral studies. GluD2 is richly expressed at dendritic spines of PCs that form synaptic contacts with terminals of parallel fibers (PFs) (Takayama et al., 1995, 1996; Landsend et al., 1997). GluD2 is essential for the formation and stabilization of PF–PC synapses by interacting with Cbln1 that binds to neurexin at PF terminals (Matsuda et al., 2010; Uemura et al., 2010). Thus, the GluD2 knockout (KO) mouse (Kashiwabuchi et al., 1995) has been shown to exhibit various defects in synaptic wiring and neuronal response: (1) the number of PF–PC synapse is reduced to nearly half of that of control mouse, which results in emergence of numerous free spines in PC distal dendrites (Kurihara et al., 1997; Ichikawa et al., 2002; Takeuchi et al., 2005), (2) CFs extend distally along PC dendrites, take over spines from PFs and form ectopic synapses on PC distal dendrites (Ichikawa et al., 2002), (3) transverse collaterals of CFs that run perpendicularly to the plane of PC dendritic tree are markedly elongated and form ectopic synapses on distal dendrites of neighboring PCs along the mediolateral axis (Miyazaki and Watanabe, 2010; Miyazaki et al., 2010), (4) stimulation of the aberrant CFs in cerebellar slices induces atypical excitatory postsynaptic responses with slow rise time and small amplitudes in PCs which are associated with calcium transients localized to distal dendritic arbors (Hashimoto et al., 2001; Miyazaki et al., 2010), and (5) PCs in GluD2 KO mice *in vivo* exhibit atypical “clustered firing (Cf)” (Yoshida et al., 2004), which is considered to be induced by ectopic CF inputs to PC distal dendrites. Thus, GluD2 KO mice provide an excellent model to study how altered CF to PC wiring affects population activity of PCs and functional microzonal organization *in vivo*.

In the present study, we employed *in vivo* two-photon calcium imaging for PC populations (Sullivan et al., 2005; Mukamel et al., 2009; Ozden et al., 2009; Schultz et al., 2009) and examined dendritic calcium signals representing CF inputs. We demonstrated that the degree of synchrony in CF inputs between neighboring PCs was much higher in GluD2 KO mice than in wild-type (control) mice. Moreover, the synchrony of CF inputs in GluD2 KO mice hardly declined with the increase in mediolateral separation between PCs, whereas the synchrony fell off within the separation of ~200 μm in control mice, which corresponded to the width of a microzone. We also showed that the enhanced synchrony in GluD2 KO mice was mainly ascribed to the aberrant CF to PC wiring, especially to elongated transverse CF collaterals, and also presumably to altered IO firing. Thus, proper formation of CF to PC wiring is a basis for functional microzonal organization in the cerebellum.

## Materials and methods

### Animals and surgery

We used homozygous Grid2-Cre knock-in mice on pure C57BL/6 genetic background (Yamasaki et al., 2011) as GluD2 knockout (GluD2 KO) mice. The GluD2 KO mice and their wild-type littermates (control) were produced by mating heterozygous animal pairs. All experimental procedures were approved by Animal Experimental Committees of The University of Tokyo and Hokkaido University, and all animal experiments were performed according to the guidelines.

Male or female mice aged 1–3 months were anesthetized by intraperitoneal injection of ketamine (100 mg/kg) and xylazine (10 mg/kg). We confirmed the depth of anesthesia by monitoring the lack of whisker movements and pinch withdrawal reflex, and injected additional dose as needed. Body temperature was kept at 36°C with a heating pad (FHC). The head of the animal was fixed by ear bars and the skull was exposed by removing skins, muscles and connective tissues on it. The occipital bone at the Crus IIa region (centered 4 mm lateral and 2 mm posterior to the occipital bone line) on the left cerebellar hemisphere was drilled to make a small hole (~2 mm in diameter). The dura matter was removed and the surface of the cerebellar cortex was cleaned with extracellular solution composed of (in mM) 150 NaCl, 2.5 KCl, 2 CaCl_2_, 1 MgCl_2_ and 10 HEPES (pH 7.4, adjusted with NaOH). Cortical surface was covered with 1.5% agarose dissolved in the extracellular solution, and a small coverslip was placed on half of the cranial window, in order to reduce motion artifacts caused by respiration and heart beat.

### Dye injection and population calcium imaging

Multi-cell bolus-loading of calcium indicator dye was performed as described (Stosiek et al., 2003; Sullivan et al., 2005; Mukamel et al., 2009; Ozden et al., 2009; Schultz et al., 2009). Oregon Green 488 BAPTA-1 acetoxymethyl ester (OGB-1 AM, ~200 μM; Invitrogen), was dissolved with 10% w/v Pluronic F-127 (Invitrogen) in DMSO and filled into a glass pipette (5–7 MΩ) together with the extracellular solution containing Alexa 594 fluorescent dye (20 μM; Invitrogen). Dye ejection was performed in the cerebellar molecular layer (50–60 μm from surface) at 5 psi for 3 min by using Picospritzer (General Valve). Successful dye ejection was monitored by two-photon imaging on Alexa channel. More than 30 min after dye ejection, calcium imaging was performed in the molecular layer. To obtain calcium transients from populations of PC dendrites, images were acquired at the resolution of 256 × 64 or 128 × 128 pixels (sampling rate = ~8 Hz) for ~2 min. These image stacks were analyzed offline. For detecting local calcium transients in bolus-loaded specimen, line-scan imaging (sampling rate = 500 Hz) was performed on single PC dendrites.

### *In vivo* two-photon microscopy

*In vivo* calcium imaging was performed by using a two-photon microscope (Denk et al., 1990) controlled by PrairieView software (Ultima IV, Prairie technologies), or a custom-built two-photon microscope (Sutter Instruments) controlled by ScanImage software (Pologruto et al., 2003). The cerebellum was illuminated with a pulsed Ti:sapphire laser (MaiTai, 810–840 nm in wavelength, 80 MHz repetition rate, 100 fsec pulse width; Spectra-Physics). Laser was focused through a 40× water-immersion objective lens (Olympus) onto the tissue. Average laser power was adjusted to be less than 20 mW at the specimen. Fluorescence signals of OGB-1 and Alexa 594 were divided into green and red channels respectively by a dichroic mirror and emission filters (Chroma), and were detected by a pair of photomultiplier tubes (Hamamatsu).

### Simultaneous dendritic calcium imaging and extracellular recording

A glass electrode (5–7 MΩ) filled with the extracellular solution containing Alexa 594 was inserted into the cerebellum and targeted to a PC soma that had been loaded with OGB-1 AM. About 10 min after the establishment of cell-attached configuration, simultaneous extracellular unit recording and dendritic calcium imaging were performed. Electrophysiological data were obtained by Multiclamp 700B (Molecular device). The data were filtered at 10 kHz and digitized at 20 kHz using Digidata 1322A (Axon instruments) controlled by Axograph X software (AxoGraph Scientific). Simple spikes (SSs), complex spikes (CSs), and Cf (Yoshida et al., 2004) were distinguished by their characteristic waveforms. CS showed a prominent spike followed by several spikelets with smaller amplitude. The burst of 2–7 full-amplitude SS-like spikes occurred at >181 Hz (spike train with <5.5 ms of inter-spike interval) was defined as Cf according to the criterion in the previous study (Yoshida et al., 2004). After the recording session, negative current (< −20 nA) was injected into the recorded cell to rupture the membrane and to stain it with Alexa 594, and a morphological image stack for Alexa 594 was obtained to identify the dendrites of the recorded cell among multiple OGB-1 positive PC dendrites in calcium imaging data.

### Drug application

Carbenoxolone (120 mg/kg; Sigma), an inhibitor of gap junction, was dissolved in saline (0.8% w/v) and intraperitoneally injected to mouse in which OGB-1 AM had been bolus-loaded. Then, calcium imaging was performed at every 20 min until 120 min after the drug application.

### Whole-cell recording and dendritic calcium imaging

For *in vivo* whole-cell current-clamp recording, we used a potassium-based intracellular solution that was composed of (in mM): 133 potassium methanesulfonate, 7.4 KCl, 10 HEPES, 3 Na_2_ATP, 0.3 Na_2_GTP, 0.3 MgCl_2_, 0.05 Alexa 594, and 0.2 OGB-1 (285 mmole/kg, pH 7.2 adjusted with KOH). According to the shadowpatching method (Kitamura et al., 2008), PCs were visualized with negative contrast to obtain targeted recordings. A glass electrode (5–9 MΩ) was brought to a PC soma under visual control and a brief suction was applied to form tight gigaohm seal. Cell membrane was then ruptured by short pulses of negative pressure to establish the whole-cell configuration. At least 30 min after break-in, line-scan calcium imaging (500 Hz) on dendrite was performed at ~100 μm from the soma and the membrane potential was simultaneously recorded.

### Data analysis

All image analysis was performed offline by using ImageJ software (http://rsb.info.nih.gov/ij/). Regions of interest (ROIs) corresponding to individual PC dendrites were manually identified. Calcium transients were obtained from time-series image stack and expressed as Δ*F*/*F* = (*F* − *F*_0_)/(*F*_0_ − *F*_*b*_), where *F*_0_ was baseline fluorescence without calcium transient and *F*_b_ was background fluorescence. The distances of mediolateral separation between PC dendrites were measured from a single high-resolution image of the recording field. The detection threshold for calcium transient was defined as 2 SD of Δ*F*/*F* for entire recording time. Cross-correlation coefficients were calculated by the formula below (Lang et al., 1996) using Igor pro software (Wavemetrics).

$$C_{ij}(\tau )=\frac{\sum_{t=0}^{T}X_{i(t)}X_{j(t+\tau )}}{\sqrt{\sum_{t=0}^{T}{\left\{X_{i(t)}\right\}}^{2}\sum_{t=0}^{T}{\left\{X_{j(t)}\right\}}^{2}}}$$

where, *X*_*i*_ and *X*_*j*_ were calcium transient traces of *i*th and *j*th dendrites, *T* was total recording time and τ was lag time between the 2 traces. *C*_*ij*_(0), cross-correlation coefficient at zero lag time, was defined as synchrony. All the averaged data were represented as mean ± s.e.m. Statistical significance of the data was examined by Mann–Whitney U test unless otherwise noted, and all tests were performed using Sigmastat 3.1 (Cranes Software International) or R (http://www.r-project.org/).

### Anterograde tracer labeling

Mice at P56 were anesthetized by intraperitoneal injection of chloral hydrate (350 mg/kg) and head-clamped by a stereotaxic instrument (SR-5N; Narishige). A glass pipette filled with 2–3 μl of 10% solution of dextran Alexa 594 (DA-594; Invitrogen) in PBS was inserted into the IO by dorsal approach. The tracer was injected by air pressure at 20 psi with 5 s intervals for 1 min (Pneumatic Picopump; World Precision Instruments). After 4 days of survival, mice were anesthetized by intraperitoneal injection of pentobarbital (100 mg/kg) and transcardially perfused with 4% paraformaldehyde in 0.1 M sodium phosphate buffer (pH 7.4). After excision from the skull, brains were further immersed overnight in the same fixative. Horizontal cerebellar sections with 50 μm thickness were prepared using a microslicer (VT1000S; Leica).

### Immunohistochemistry

Horizontal cerebellar sections were obtained from 3 GluD2 KO and 3 control mice. All immunohistochemical incubations were done at room temperature in a free-floating state. At first, cerebellar sections were incubated with 10% normal donkey serum for 20 min. Then, a mixture of primary antibodies, including a guinea pig anti-vesicular glutamate transporter 2 (VGluT2) antibody and a rabbit anti-aldolase C (aldC) antibody, was applied to slices overnight, followed by an incubation with Alexa 488- and Cy5-conjugated species-specific secondary antibodies (Invitrogen; Jackson ImmunoResearch) for 2 h at a dilution of 1:200. Images of stained molecular layer were taken with a confocal laser scanning microscope (FV1000; Olympus) equipped with digital camera (DP70; Olympus), and analyzed with MetaMorph software (Molecular Devices).

## Results

### Highly synchronous spontaneous calcium transients in neighboring PCs in GluD2 KO mice

**Figure 1:**
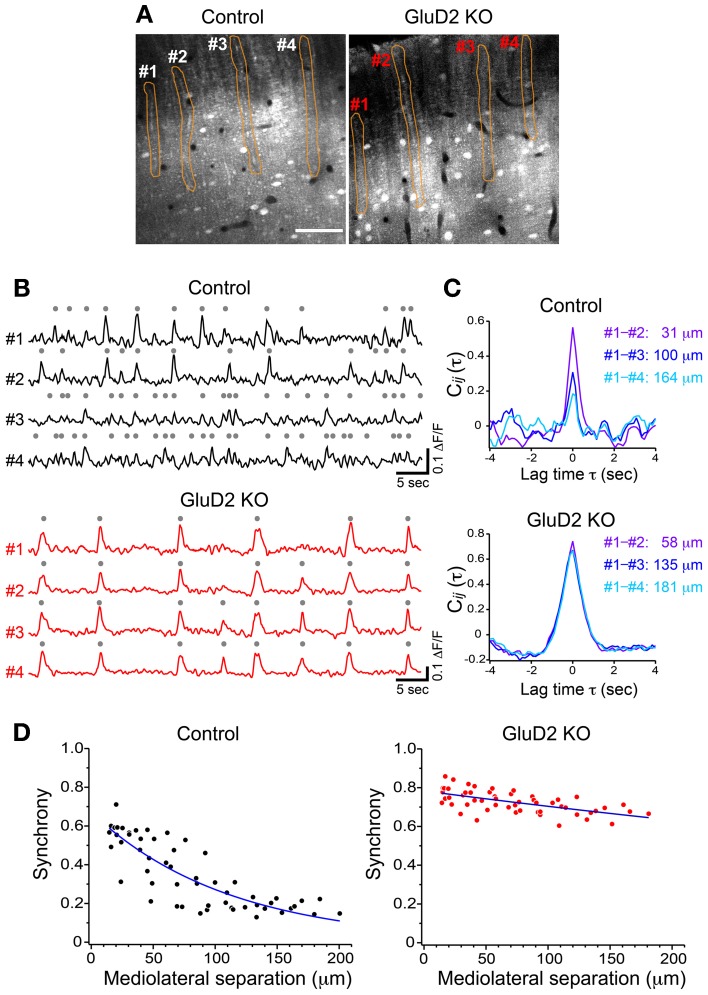
**Synchrony of spontaneous calcium transients in control and GluD2 KO mice. (A)** Fluorescence images showing the molecular layer of the cerebellar cortex labeled with OGB-1 AM in control and GluD2 KO mice. PC dendrites are seen as gray bands that extend along the rostrocaudal axis and aligned perpendicular to the mediolateral axis. Spontaneous calcium transients were obtained by monitoring fluorescence changes of the specified regions of interest (ROIs; orange). Scale bar, 50 μm. **(B)** Representative traces of calcium transient from 4 dendrites (#1 – #4) that correspond to the ROIs in **(A)**. Gray dots indicate detected calcium transients from the baseline noise. **(C)** Cross-correlation coefficients for the traces in **(B)** were calculated between #1 and the other dendrites, and the distances along the axis (mediolateral separation) between the dendrites were measured. Synchrony (i.e., the cross-correlation at zero lag time, see Materials and Methods) in control mice was the highest at the nearest neighboring pair, and gradually decreased as the mediolateral separation increased. In marked contrast, synchrony in GluD2 KO mice was almost constant regardless of the mediolateral separation. **(D)** Summary graphs for the synchrony between all dendrite pairs plotted against the mediolateral separations from the control and GluD2 KO mice shown in **(A)** (55 dendrite pairs). Blue curves indicate the exponential fit to the data.

**Figure 2:**
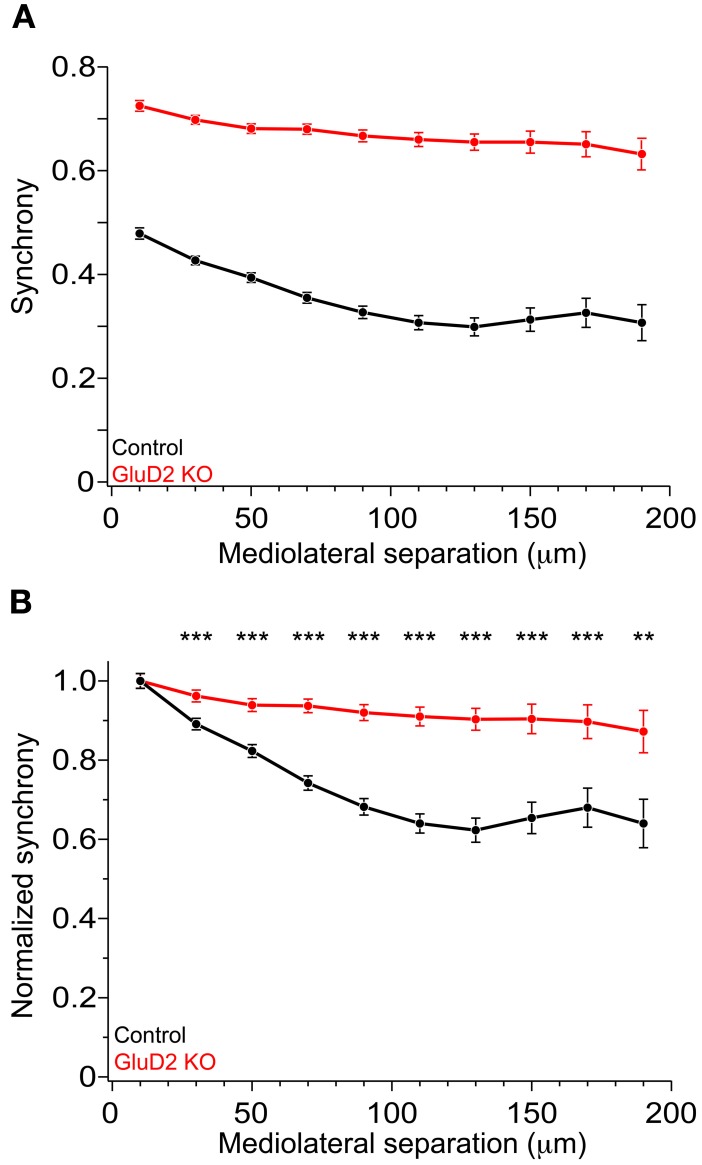
**Enhanced synchrony between dendrite pairs of GluD2 KO mice at all the distances of mediolateral separation. (A)** Summary graph showing pooled data of synchrony plotted against the mediolateral separation for 1009 dendrite pairs obtained from 33 control mice (black symbols) and for 1064 dendrite pairs from 26 GluD2 KO mice (red symbols). Each data point represents the average of synchrony and error bars indicate SEM. There was significant difference between the two genotypes (*p* < 0.001 by Two-Way ANOVA). **(B)** All values of synchrony were normalized by the mean value of the nearest dendrite pairs within the mediolateral separation of 20 μm. There were significant differences between the two genotypes in all but the nearest pairs (^**^p < 0.01, ^***^p < 0.001 by Two-Way ANOVA and Tukey test), indicating that the degree of decline in synchrony in the mediolateral direction is much smaller in GluD2 KO mice than that in control mice.

In rats and mice, spontaneous calcium transients in PC dendrites *in vivo* have been shown to be attributed to CSs evoked by CF input (Sullivan et al., 2005; Ozden et al., 2008, 2009; Mukamel et al., 2009; Schultz et al., 2009; Kitamura and Häusser, 2011). To compare the spatial pattern of CS firing in PCs of GluD2 KO mice with that of wild-type control mice, OGB-1 AM was bolus-loaded into the molecular layer of the cerebellar cortex (Ozden et al., 2008, 2009; Mukamel et al., 2009; Schultz et al., 2009). About 30 min after dye-loading, OGB-1 was penetrated into various cell types, including PCs, interneurons, and Bergmann glia. In the molecular layer, dendrites of PCs extending along rostrocaudal axis were clearly observed (Figure 1A). Spontaneous fluorescence changes induced by calcium influx were observed in individual PC dendrites. In control mice, calcium transients occurred at various timing in each dendrite with occasional highly synchronized transients between neighboring dendrites (Figure 1B, upper), which is consistent with the previous results (Sullivan et al., 2005; Ozden et al., 2008, 2009; Mukamel et al., 2009; Schultz et al., 2009). In contrast, almost all calcium transients in GluD2 KO mice occurred at the same timing in all of the dendrites observed (Figure 1B, lower). Cross-correlation coefficient was calculated to quantify synchronous activity between neighboring PCs (Figure 1C), and the relationship between the synchrony and the distance between the dendrites in mediolateral direction was analyzed for all dendritic pairs in the field of view (Figure 1D). As previously reported, synchrony in control mice fell off as the mediolateral separation between PC dendrites increased (Ozden et al., 2009; Schultz et al., 2009). In marked contrast, the synchrony in GluD2 KO mice was almost constant at all the distances of mediolateral separation examined. On average, the synchrony in GluD2 KO mice was significantly higher than that in control mice at all the distances of mediolateral separation (1064 dendrite pairs in 26 GluD2 KO mice and 1009 pairs in 33 control mice, *p* < 0.001 in Two-Way ANOVA) (Figure 2A), and the rate of decline in synchrony was smaller in GluD2 KO mice than in control mice (Figure 2B). These results indicate that the synchrony of spontaneous CSs among neighboring PCs is greatly enhanced in GluD2 KO mice, and the spatial range of synchrony is extended in mediolateral direction.

### Relationship between calcium transient and CF input in GluD2 KO mice

**Figure 3:**
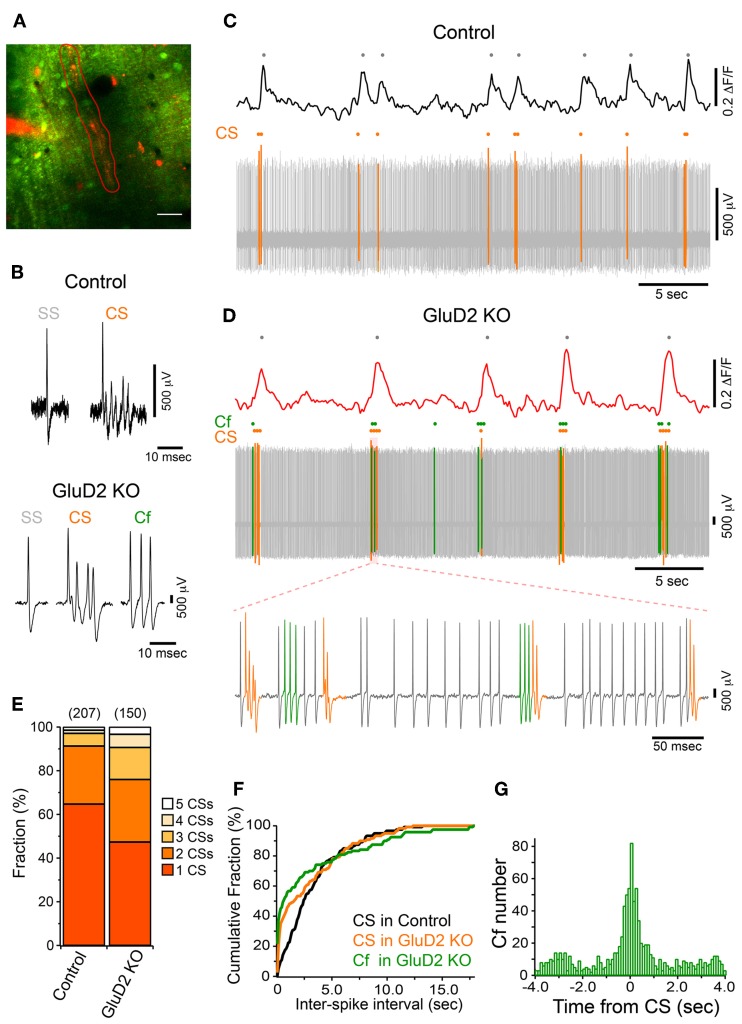
**Altered CF activity patterns in GluD2 KO mice. (A)** Representative image showing the molecular layer that was loaded with OGB-1 AM in a GluD2 KO mouse. Simultaneous cell-attached recording and dendritic calcium imaging from single PCs were performed. After the recording, Alexa 594 was injected through the patch pipette to the PC from which cell-attached recording was conducted. Spontaneous calcium transients were obtained from the ROI enclosed by red line. Scale bar, 20 μm. **(B)** Sample traces for simple spike (SS), complex spike (CS), and clustered firing (Cf) under cell-attached recordings from control (upper panel) and GluD2 KO (lower panel) mice. Cf was not observed in control mice. **(C)** Simultaneous recording of PC firing and dendritic calcium transients in a control PC. Note that all the calcium transients are associated with CSs. **(D)** Simultaneous recording of PC firing and dendritic calcium transients in the PC shown in **(A)**. SS, CS, and Cf are indicated in gray, orange, and green, respectively. Note that calcium transients are elicited only when CS occurs, or when CS and Cf fire in cluster. The bottom trace shows clustered CS and Cf in an expanded timescale. In this period, a single calcium transient included multiple CSs and Cfs that fired in temporal proximity. **(E)** Bar chart showing the proportion of calcium transient induced by 1–5 CSs. The fraction of each component was significantly different between control and GluD2 KO mice (4 mice for each genotype; *p* < 0.001 by χ^2^ test). The numbers in parentheses indicate the number of calcium transients. **(F)** Cumulative probability plot of inter-spike intervals for CS and Cf. Each curve is composed of 120 intervals obtained from 6 mice for each genotype. Curves for CS and Cf in GluD2 KO mice were significantly different from the curve for CS in control mice (*p* < 0.001 by K-S test). Moreover, there was a significant difference between the curve for CS and that for Cf in GluD2 KO mice (*p* = 0.004 by K-S test). Bin width, 100 ms. **(G)** Cross-correlogram of Cf against CS was constructed using the data from 8 GluD2 KO mice. Note that a prominent peak is present at 0 s. Bin width, 25 ms.

In addition to the enhancement of the synchrony between calcium transients of neighboring PCs, we found that the frequency of calcium transient in GluD2 KO mice was lower than that in control mice (0.15 ± 0.01 Hz and 0.29 ± 0.02 Hz, respectively; 11 mice each, *p* < 0.001) (Figure 1B), and the half-width of transients was larger in GluD2 KO mice than in control mice (0.710 ± 0.01 s and 0.517 ± 0.01 s, respectively; 5 mice each, *p* < 0.001), suggesting that the temporal pattern of CS firing is altered in GluD2 KO mice. Therefore, simultaneous dendritic calcium imaging and extracellular recordings were performed on single PCs to clarify the electrophysiological correlates of calcium transients (Figure 3A). Cell-attached recordings in both control and GluD2 KO mice showed ongoing SSs and sporadic CSs (Figure 3B). The mean firing rates of SS (22.12 ± 5.39 Hz in control and 17.19 ± 2.33 Hz in GluD2 KO mice) and CS (0.32 ± 0.04 Hz in control and 0.40 ± 0.03 Hz in GluD2 KO mice) were not significantly different between the two genotypes (13 mice each; *p* > 0.09 and *p* > 0.1, respectively). Although calcium transients were induced by CS in both genotypes (Figures 3C,D), each calcium transient in GluD2 KO mice tended to be associated with multiple successive CSs. The fraction of calcium transients induced by multiple CSs was significantly higher in GluD2 KO mice than in control mice (Figure 3E, *p* < 0.001 in χ^2^ test). Besides, the histogram of inter-CS interval clearly showed that CSs in GluD2 KO mice were induced in rapid succession with short interval (Figure 3F). These results indicate that the lower frequency and longer duration of calcium transients in GluD2 KO mice were attributed to the altered CS firing pattern.

Atypical responses, termed “Cf” (Yoshida et al., 2004), were observed only in GluD2 KO mice at similar frequency to CS firing rate (0.39 ± 0.09 Hz in 11 mice; *p* > 0.1; see Materials and Methods for the definition of Cf). Cf was thought to be induced by aberrant CF input (Yoshida et al., 2004). Cfs showed similar or even shorter inter-Cf intervals than CS (Figure 3F). Moreover, most Cfs occurred temporally close to CS (Figures 3D,G). These results suggest that the firing pattern of IO neurons is altered in GluD2 KO mice such that bursts of CSs/Cfs frequently occur in PCs.

### The effect of gap junctional coupling on enhanced synchrony of CS activity in GluD2 KO mice

**Figure 4:**
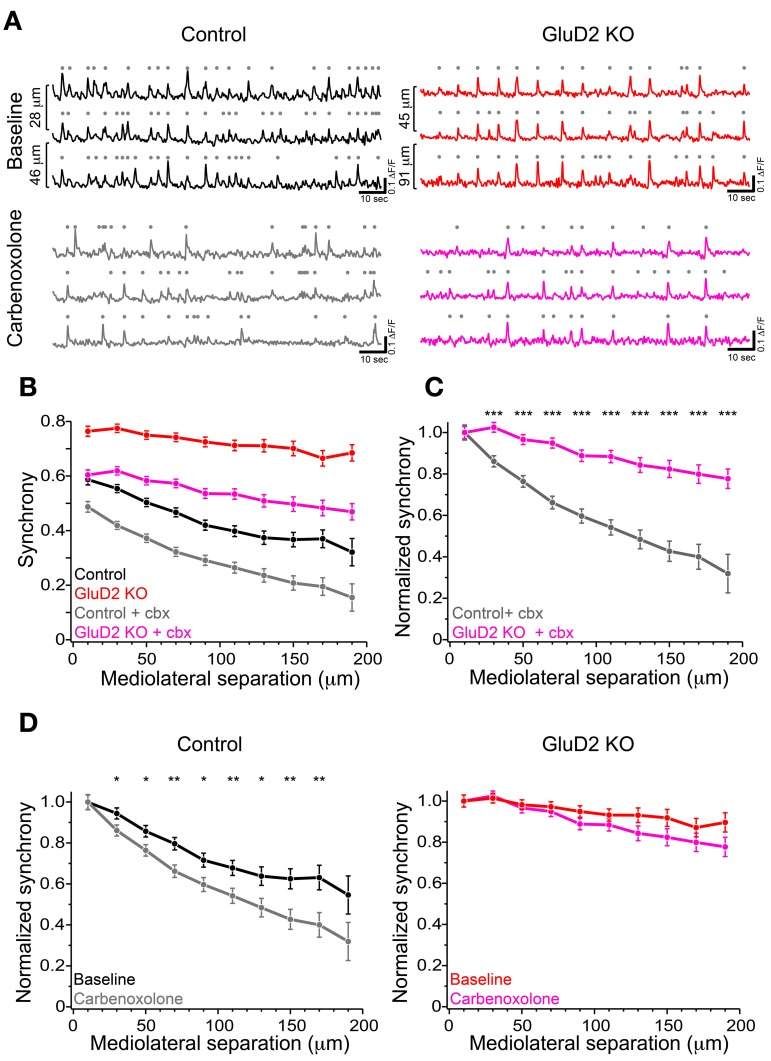
**Carbenoxolone reduced the synchrony but little altered the degree of decline along the mediolateral axis in GluD2 KO mice. (A)** Three representative traces of calcium transient from neighboring PC dendrites before (baseline) and ~1 h after carbenoxolone injection (carbenoxolone), obtained from control (left) and GluD2 KO (right) mice. Distances of mediolateral separation between dendrites are indicated on the left of the traces. In both genotypes, synchronous calcium transients were apparently reduced in frequency by carbenoxolone. **(B)** Summary graph showing pooled data of synchrony for 318 dendrite pairs obtained from 8 control mice and for 267 dendrite pairs from 5 GluD2 KO mice. Carbenoxolone (cbx) injection reduced mean values of synchrony in both genotypes. It should be noted that the difference between the genotypes was highly significant after carbenoxolone injection [control + cbx (gray) vs. GluD2 KO + cbx (magenda), *p* < 0.001 by Two-Way ANOVA], as was before drug application (control (black) vs. GluD2 KO (red), *p* < 0.001 by Two-Way ANOVA). **(C)** Normalized synchrony curves of control and GluD2 KO mice after carbenoxolone injection. The rate of decline against the mediolateral separation was significantly smaller in GluD2 KO mice than that in control mice (^***^p < 0.001 by Two-Way ANOVA and Tukey test). **(D)** Normalized synchrony curves before and after carbenoxolone injection plotted for control (left graph) and GluD2 KO (right graph) mice. In control mice, synchrony was significantly decreased by carbenoxolone at all but the nearest and the farthest points of separation, whereas in GluD2 KO mice, significant differences were not detected at all points of mediolateral separation points (^*^p < 0.05, ^**^p < 0.01 by Two-Way ANOVA and Tukey test).

According to previous reports, altered modulatory inputs in the IO by pharmacological manipulation causes change in rhythmicity and synchrony of CS firing (Llinas and Sasaki, 1989; Lang et al., 1996; Lang, 2002). To examine whether the change in electrical coupling among IO neurons made significant contribution to the enhanced synchrony of CS firing in GluD2 KO mice, carbenoxolone, a non-selective blocker of connexin, was intraperitoneally injected during calcium imaging experiments. Although systemic application of carbenoxolone has been reported to cause various effects on whole mouse body (Rozental et al., 2007), a previous study demonstrates that it has no effects on PC firing (Cheron et al., 2004). About 1 h after drug application, synchronized calcium transients were reduced in both control and GluD2 KO mice (Figures 4A,B). As a result, the average synchrony significantly decreased (from 0.48 ± 0.04 to 0.34 ± 0.02 in 8 control, *p* = 0.002 by paired *t*-test; from 0.73 ± 0.03 to 0.55 ± 0.04 in 5 GluD2 KO mice, *p* = 0.003). The degree of reduction in the synchrony by carbenoxolone injection was not significantly different between the two genotypes (0.14 ± 0.03 in control and 0.18 ± 0.03 in GluD2 KO mice; *p* > 0.1). Therefore, the values of synchrony at all the distances of mediolateral separation in GluD2 KO mice were still larger than those in control mice even though electrical couplings in IO neurons were inhibited (Figure 4B; *p* < 0.001 by Two-Way ANOVA). When the synchrony values were normalized to those within 20 μm separation, the rate of decline in the synchrony along the mediolateral axis remained much smaller in GluD2 KO mice than in control mice after carbenoxolone application (Figure 4C). While carbenoxolone enhanced the rate of decline in the synchrony along the mediolateral axis to some extent in control mice, it had much smaller effect in GluD2 KO mice (Figure 4D). These results indicate that the enhancement of synchrony in the mediolateral direction is likely to be attributable largely to mechanisms other than gap junctional coupling in IO neurons.

### Multi-zonal projection of transverse branches from ascending CF in GluD2 KO mice

We hypothesized that the enhanced mediolateral synchrony in GluD2 KO mice was due to disorganized olivo-cerebellar projection by the persistent multiple CF innervation (Kashiwabuchi et al., 1995; Hashimoto et al., 2001; Ichikawa et al., 2002; Miyazaki et al., 2010). Immunohistochemical labeling of CF terminals combined with tracer injection into the IO has revealed that dendrites of PCs in GluD2 KO mice make contact with glutamatergic terminals from ascending CFs as well as from transverse collaterals of neighboring ascending CFs (Ichikawa et al., 2002; Miyazaki et al., 2010). Thus, these aberrant transverse branches were assumed to contribute to the enhanced mediolateral synchrony beyond the proper range of microzone in GluD2 KO mice. To elucidate whether CFs projecting to a microzone could extend their transverse branches to neighboring microzones in GluD2 KO mice, we examined the relationship between the extension of CF transverse branches and the expression pattern of aldolase C (aldC) in PCs. AldC is expressed in PCs aligned in longitudinal stripes (Hawkes and Leclerc, 1987; Brochu et al., 1990; Sugihara and Quy, 2007), and the tight link between aldC compartments and CF projections and CS synchrony have been shown in previous studies (Voogd et al., 2003; Sugihara and Shinoda, 2004; Voogd and Ruigrok, 2004; Pijpers et al., 2005; Sugihara et al., 2007).

**Figure 5:**
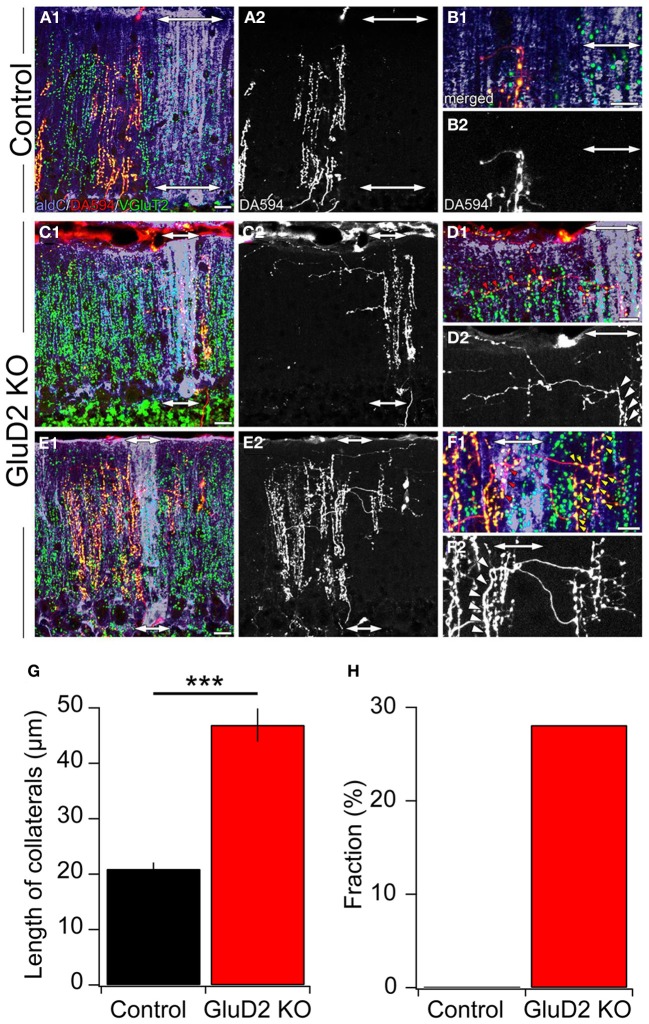
**Ectopic CF innervations beyond the zones in GluD2 KO mice. (A)** Photomicrographs of a horizontal section of cerebellar cortex from a control mouse. In **(A1)**, aldC (purple) and VGluT2 (green) positive structures are present beside CFs labeled with DA594 (red). Double-headed arrows indicate the region in which aldC-positive PCs are localized. In this case, the tracer was taken up by CFs projecting to an aldC-negative zone. **(A2)** is the same section as **(A1)** but shows DA594-labeled CFs only. **(B)** Magnified pictures of the outer molecular layer near the pial surface in **(A1)** and **(A2)**. There are short transverse CF collaterals that lack VGluT2-positive puncta and do not reach the adjacent aldC-positive zone. **(C)** Photomicrographs of a horizontal section of cerebellar cortex from a GluD2 KO mouse. In this case, transverse CF collaterals bifurcate from ascending CFs that project to aldC-positive PCs. Note that transverse CF collaterals extend toward the adjacent aldC-negative zone. **(D)** Magnified pictures of the outer molecular layer near the pial surface in **(C1)** and **(C2)**. Red arrowheads in **(D1)** indicate VGluT2-positive glutamatergic terminals of transverse CF collaterals that form synaptic contacts onto multiple PCs. White arrowheads in **(D2)** indicate the origins of transverse CF collaterals. **(E)** Photomicrographs of a horizontal section of cerebellar cortex in another GluD2 KO mouse. In this case, transverse CF collaterals bifurcate from ascending CFs that innervate aldC-negative PCs and extend toward the next aldC-negative zone beyond the adjacent aldC-positive zone. **(F)** Magnified pictures of the outer molecular layer near the pial surface in **(E1)** and **(E2)**. Red and yellow arrowheads in **(F1)** indicate VGluT2-positive glutamatergic terminals of transverse CF collaterals that form numerous synaptic contacts onto aldC-positive and aldC-negative PCs, respectively. White arrowheads in **(F2)** indicate the origins of transverse CF collaterals. Scale bar, 20 μm in **(A,C,E)**; 10 μm in **(B,D,F)**. **(G)** Length of transverse CF collaterals was significantly longer in GluD2 KO mice than in control mice (^***^p < 0.001). **(H)** Fraction of transverse branches, which had numerous excitatory terminals to broad region of PC dendrites in the neighboring zones.

A small amount of dextran Alexa 594 (DA594) was injected into a certain subnucleus of the IO in order to visualize the trajectories of subsets of CFs and their innervation patterns. After 4 days of survival, mice were sacrificed, brains were removed and cerebellar sections were prepared. The sections were immunostained for aldC, type 2 vesicular glutamate transporter (VGluT2, a CF terminal marker) and DA594. In control mice, ascending CFs had a few short transverse branches (Figures 5A2,B2) that had no detectable VGluT2-positive puncta (Figures 5A1,B1). These short processes (20.9 ± 1.2 μm; 3.6–107.5 μm; *n* = 188) originated from an aldC-negative zone and did not reach the neighboring aldC-positive zone (Figures 5A,B), and vice versa. By contrast, much longer transverse branches (Figure 5G; 46.9 ± 3.0 μm; 5.3–176.8 μm; *n* = 135, *p* < 0.001) containing many VGluT2-positive puncta were observed in GluD2 KO mice (Figures 5C–F). These branches bifurcated from ascending CFs located in an aldC-positive (Figures 5C,D) or -negative (Figures 5E,F) zone, and elongated toward neighboring zones. Furthermore, they made synaptic connections with multiple PCs aligned in the mediolateral axis beyond the border of aldC compartments (Figures 5D,F). Notably, 28.1% (38/135) of total transverse branches in GluD2 KO mice had numerous excitatory terminals to broad region of PC dendrites in the neighboring zones (Figure 5F; red and yellow arrowheads), whereas no such branch was observed in control mice (0/188) (Figure 5H). These morphological data strongly suggest that PCs within a certain microzone receive inputs from ascending CFs in that microzone and from CF transverse collaterals originating from neighboring microzones in GluD2 KO mice.

### Calcium transients in distal dendrites induced by aberrant CF inputs in GluD2 KO mice

**Figure 6:**
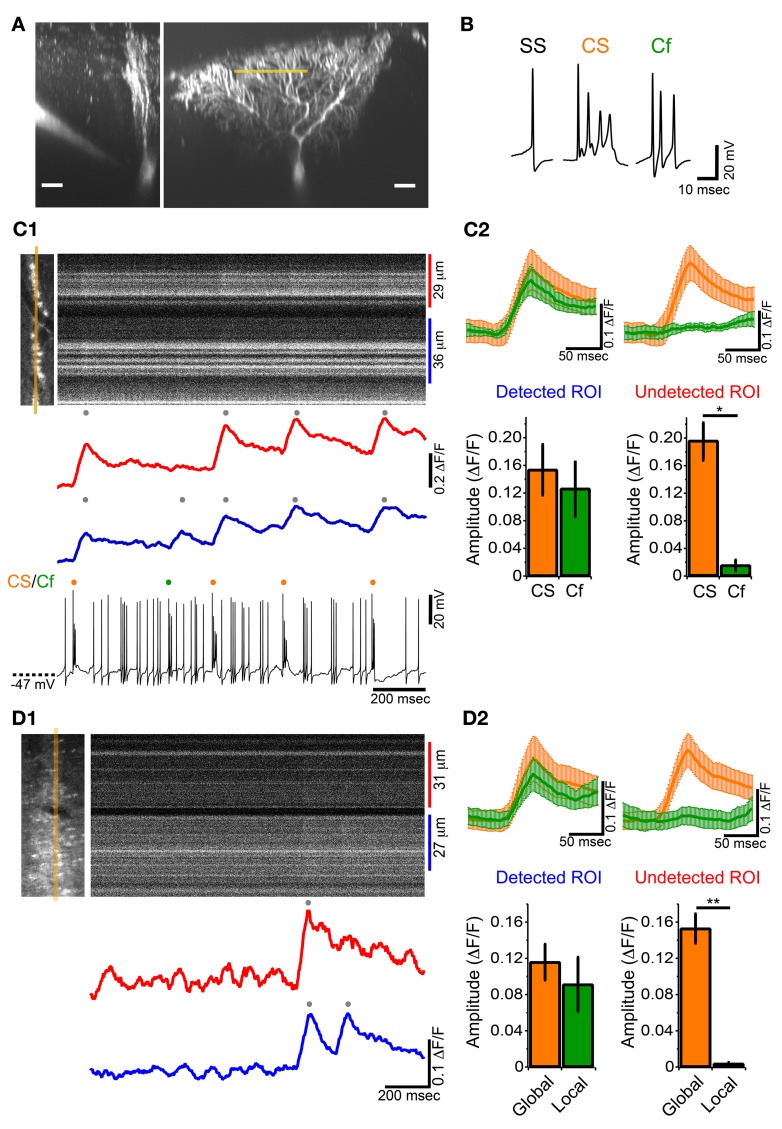
**Local calcium transient induced by Cf in GluD2 KO mice. (A)** Fluorescence images showing a whole-cell patch-clamped PC of a GluD2 KO mouse in transverse (left) and sagittal (right) views that were taken more than 30 min after filling of OGB-1 and Alexa 594 through the patch pipette. Line-scan calcium imaging was performed at distal dendrite (~100 μm from the soma) indicated by orange line. Scale bar, 20 μm. **(B)** Sample traces of simple spike (SS), complex spike (CS), and clustered firing (Cf) under current-clamp recording from a GluD2 KO mouse. **(C1)** Simultaneous whole-cell recording and line-scan calcium imaging was obtained from the PC shown in **(A)**. Left image indicates the location of line-scan (orange line). ROIs were determined so that local calcium transients could be maximally recorded. The blue trace was obtained from the lower ROI in which local calcium transient was recorded, and the red trace was obtained from the upper ROI in which no local calcium transients were detected for Cf. The onset of local calcium transient coincided with the time when Cf occurred. **(C2**) (upper panels) Average traces of calcium transients induced by CS (orange trace) and by Cf (green trace) in the ROI from which Cf-evoked local calcium transients were detected (Detected ROI) and in the other ROI of the same PC in which Cf-evoked local calcium transients were absent (Undetected ROI). CS- or Cf-evoked calcium transients are shown as mean ± s.e.m from 4 GluD2 KO mice. Note that the magnitude and time course of Cf-evoked calcium transient in the detected ROI was comparable to those of CS-evoked calcium transient (lower panels). Bar graphs showing the magnitudes of calcium transients in the detected ROI (left) and undetected ROI (right). There was no significant difference in magnitude between CS-evoked and Cf-evoked calcium transients in the detected ROI (left, *p* > 0.1). By contrast, there was no detectable fluorescence change in the undetected ROI when Cf occurred (right; ^*^p = 0.03). **(D1)** Line-scan imaging on a PC distal dendrite from a GluD2 KO mouse that was stained by bolus-loading of OGB-1 AM. Left picture indicate the location for line-scan (orange line). A local calcium transient was detected in the lower ROI (blue trace) but was absent in the upper ROI (red trace). **(D2)** Average traces of calcium transients (upper panels, from 5 GluD2 KO mice) and bar graphs showing the magnitudes of global and local calcium transients (lower panels) for the ROI from which local calcium transients were recorded (Detected ROI) and for the ROI in which local calcium transients were absent (Undetected ROI). Data are illustrated similarly to **(C2)**. There was no significant difference in magnitude between global and local calcium transients in the detected ROI. There was no detectable fluorescence change in the undetected ROI (right; ^**^p = 0.008) at the time when local calcium transients occurred in the detected ROI.

To examine whether ectopic CF branches, including transverse branches can induce detectable calcium transient *in vivo*, simultaneous somatic whole-cell recordings and calcium imaging at distal dendrite were performed on single PCs in GluD2 KO mice (Figure 6A). SS, CS and Cf were detected in current-clamp recordings (Figure 6B). High-speed line-scan imaging (2 ms/line) was performed to detect individual calcium transients, because half of the CSs and Cfs occurred with subsecond inter-spike interval (Figure 3F). CSs induced calcium transients over the entire dendritic arbor (Figures 6C1,C2—orange traces and columns), which was the same as in control mice and in rats (Kitamura and Häusser, 2011). By contrast, Cfs induced calcium transients only at a restricted region of distal dendrites (Figure 6C). As exemplified in Figure 6C, Cf-evoked transients were consistently observed only in the lower dendritic region (Figures 6C1,C2—left panel, green trace and column) and virtually no calcium signals were detected in the upper dendritic region (Figures 6C1,C2—right panel, green trace and column). This result is very similar to that obtained in cerebellar slice preparations from GluD2 KO mice (Hashimoto et al., 2001) in which stimulation of surplus weak CFs induced calcium transients restricted to small regions of distal dendritic arbors of PCs. It should be noted that, although Cf-evoked calcium transients were spatially localized, no significant difference was found between the amplitudes of calcium transients evoked by Cf and those by CS in the same dendritic regions (Figure 6C2, left panel). Importantly, Cfs and localized calcium transients were not detected in control mice (11 mice).

We also found that localized calcium transients could be detected in PC dendrites of GluD2 KO mice that were filled with OGB-1 AM by bolus-loading method (Figure 6D). In the case shown in Figure 6D1 and upper panel of Figure 6D2, a calcium transient that was detected in both the upper and lower dendritic regions (Figure 6D1, red and blue traces; Figure 6D2 upper panel, orange traces) was followed by another calcium transient that was confined to the lower dendritic region (Figure 6D1, blue trace; Figure 6D2 upper panel, green traces). The summary data demonstrated that global and localized calcium transients were present in PC dendrites of GluD2 KO mice (Figure 6D2 lower panel). The global and localized calcium transients of PCs in GluD2 KO mice by bolus-loading of OGB-1 AM (Figure 6D) were very similar to CS-evoked and Cf-evoked calcium transients, respectively, in whole-cell recorded PCs in GluD2 KO mice (Figure 6C). Since aberrant CF inputs to PCs in GluD2 KO mice are thought to induce Cfs in intact cerebellum (Yoshida et al., 2004) and local calcium transients in cerebellar slices (Hashimoto et al., 2001), these results indicate that the localized calcium transients were induced, at least in part, by aberrant CF inputs from transverse branches. The amplitudes of these localized calcium transients were comparable to those of the global calcium transients (Figures 6C2,D2). Therefore, the localized calcium transients elicited by CF transverse collaterals are likely to contribute to the enhanced synchrony of PC activity along the mediolateral axis.

## Discussion

In the present study, we demonstrated that PCs within a given microzone in GluD2 KO mice showed higher synchrony of CF activities than in control mice, and the synchrony remained high even when the mediolateral separation increased. The enhancement of synchrony in mediolateral direction was ascribed to the local calcium transients evoked by Cfs that frequently occurred in close succession to the global calcium transients evoked by CSs. These local calcium transients were likely to be induced by aberrant CF branches, including transverse collaterals elongated along the mediolateral axis beyond the border of cerebellar zones.

### Origin of the mediolateral enhancement of CS synchrony in GluD2 KO mice

It has been demonstrated that surplus weak CF input can induce localized calcium transients in distal dendrites of PCs in cerebellar slice preparations from GluD2 KO mice (Hashimoto et al., 2001). A recent morphological study also showed that ectopic CF branches in GluD2 KO mice innervate limited areas in the distal dendritic tree of PCs, and that transverse CF collaterals elongating in the outer molecular layer near the pial surface have glutamatergic terminals around dendrites of multiple neighboring PCs (Miyazaki and Watanabe, 2010). These elongated transverse CF collaterals and ectopic synapse formation occur mutually among neighboring PCs (Miyazaki and Watanabe, 2010). Thus, CFs of GluD2 KO mice are considered to activate their main target PCs through ascending main branches and also neighboring PCs through transverse collaterals. Spike discharges of IO neurons in GluD2 KO mice can induce global dendritic calcium transients associated with CSs in their main target PCs, and also induce local dendritic calcium transients associated with Cfs in neighboring PCs (Figure 6). Furthermore, we found that Cfs tended to be induced in a rapid succession (Figure 3F) in close proximity to the occurrence of CS (Figure 3G). At a whole PC level, bursts of Cfs are triggered by several aberrant branches from surrounding CFs and can induce local calcium transients in various dendritic regions of the same PC simultaneously. These clustered “local” calcium signals are closely correlated in time with the CS-evoked “global” calcium signals observed in other neighboring PCs, and thus significantly contribute to the enhanced synchrony in the mediolateral direction.

In contrast to the aberrant CF–PC wiring, the gap junctional coupling between IO neurons seemed less important for the enhanced mediolateral synchrony of CF activity in GluD2 KO mice (Figures 4C,D). However, the synchrony between adjacent PCs (distance between dendrites = 20 μm) of GluD2 KO mice was reduced to the level of control mice by application of a gap junction blocker (Figure 4B). This result indicates that the gap junctional coupling among IO neurons contributes significantly to the enhanced synchrony among adjacent PCs in GluD2 KO mice.

### Change in firing patterns of PCs and its possible impacts on activities of the olivo-cerebellar loop in GluD2 KO mice

We found that not only spatial pattern but also temporal pattern of CF activity was altered in GluD2 KO mice (Figure 3) as reported previously (Yoshida et al., 2004). This alteration in firing pattern was also important for the enhanced synchrony in mediolateral direction. Previous studies demonstrated that firing patterns of IO neurons are modulated by glutamatergic and GABAergic inputs (Lang et al., 1996; Lang, 2002). These inputs are elements of the olivo-cerebellar feedback loop composed of IO, PCs and deep cerebellar nuclei (DCN). IO neurons send their axons to PCs in specific cerebellar zones and their axon collaterals to specific groups of DCN neurons that receive inhibitory inputs from the same PCs to which the same IO neurons project. A part of DCN neurons in turn send inhibitory signals back to the IO (De Zeeuw et al., 1989; Lang et al., 1996; Chen et al., 2010). On the other hand, another population of DCN neurons send excitatory inputs to mesodiencephalic nuclei, such as the red nucleus and the nucleus of Darkschewitsch, which send excitatory signals back to the IO (De Zeeuw et al., 1998). Our results suggest that these excitatory and inhibitory inputs to the IO are likely to be altered by the change in spatial and temporal patterns of CF responses of PCs in GluD2 KO mice. In addition to typical CF response (CS), PCs in GluD2 KO mice showed a characteristic burst of action potentials (Cf), which is most likely due to surplus weak CF inputs arising from aberrant transverse CF collaterals (Yoshida et al., 2004). A Cf is a burst of 2–7 full spikes at a firing rate of >180 Hz, whereas a CS consists of a single full spike followed by several smaller spikelets (Figure 3B). As previously shown by simultaneous somatic and axonal recordings from PCs in cerebellar slice preparations, PC axons can transmit SSs with high fidelity at >200 Hz, whereas they cannot faithfully transmit the secondary spikelets in CS (Khaliq and Raman, 2005; Monsivais et al., 2005). Therefore, weak surplus CF inputs, which induce Cfs, would have significant or even larger impact on the output of PC to DCN, compared with strong main CF inputs that induce typical CSs. Furthermore, CS and Cf in GluD2 KO mice fired in burst (2–16 CS/Cf at 6–12 Hz), even though the mean firing rate was not significantly different from the CS firing rate of control mice. Given the highly convergent nature of connections between PCs and DCN neurons (Palkovits et al., 1977), spatial enhancement of synchrony and temporal clustering of CF activity in PCs would have striking effects on the firings of DCN neurons in GluD2 KO mice. Since synchronized CSs in PCs within a microzone can induce strong hyperpolarization followed by rebound spiking in corresponding DCN neurons (Bengtsson et al., 2011), DCN neurons in GluD2 KO mice are likely to show strong rebound burst firings in response to synchronized CS/Cf clusters. Such burst firings of DCN neurons may generate burst spiking in IO neurons through the excitatory pathway involving mesodiencephalic nuclei, which may eventually be converted to CS bursts in PCs via CFs. On the other hand, burst firings of DCN neurons that send inhibitory inputs directly to the IO are thought to induce large hyperpolarization and rebound excitation in IO neurons (Khosrovani et al., 2007; Choi et al., 2010). Thus, it is expected that highly synchronized PC activation by CS/Cf causes strong modulation of the firing patterns of IO neurons through rebound excitation of DCN neurons in GluD2 KO mice.

### Highly synchronous CF activity between PCs in GluD2 KO mice

Previous studies has revealed various cytoarchitectural, electrophysiological and behavioral phenotypes of GluD2 KO mice, including impaired PF–PC synapse formation (Kurihara et al., 1997; Ichikawa et al., 2002; Takeuchi et al., 2005), persistent multiple CF innervation (Hashimoto et al., 2001; Ichikawa et al., 2002; Miyazaki et al., 2010), deficient PF-LTD (Kashiwabuchi et al., 1995; Hirai et al., 2005a), abnormal rhythmic CF firing (Yoshida et al., 2004), ataxic gait (Kashiwabuchi et al., 1995; Hirai et al., 2005a), oscillatory eye movements (Yoshida et al., 2004) and impaired motor learning (Kashiwabuchi et al., 1995; Yoshida et al., 2004; Hirai et al., 2005a). In addition to these deficits, the present study has revealed a novel phenotype of GluD2 KO mice that the synchrony of CF responses among neighboring PCs are greatly enhanced in the mediolateral direction of the cerebellar cortex (Figures 1, 2). Parasagittal longitudinal microzones based on the olivo-cerebellar projection are the functional units of the cerebellar cortex and play a central role in information processing in the cerebellum (Buisseret-Delmas and Angaut, 1993; Sugihara and Shinoda, 2004). Sensory and/or motor information can be robustly encoded in spatial patterns of activated microzones (Welsh et al., 1995; Ozden et al., 2009; Schultz et al., 2009; Ghosh et al., 2011), and the synchrony of CF activities among neighboring PCs in a given microzone can convey sensorimotor information without increasing the firing rates of CFs (Welsh, 2002; Schultz et al., 2009; Bosman et al., 2010). In GluD2 KO mice, the abnormal enhancement of CF synchrony in the mediolateral direction leads to functional broadening of individual microzones and reciprocal reduction in the pattern of activated microzones. This should significantly reduce the quantity of information conveyed by CF inputs, which may result, at least in part, in motor deficits in GluD2 KO mice.

### Disrupted zonal organization by aberrant transverse CF collaterals in GluD2 KO mice

According to the previous morphological studies, CF terminals of single IO neurons are aligned within single aldC bands, and these compartments are thought to represent functional units (“zones”) for motor control of specific body parts (Sugihara and Shinoda, 2004). The rate of decline in synchrony in GluD2 KO mice was smaller than that in control mice (~10% decrease and 35–45% decrease at 190 μm, respectively; Figures 2B, 4D), indicating that PCs in different zones of GluD2 KO mice could be activated simultaneously. In fact, transverse CF collaterals branching from ascending CFs in an aldC-positive band elongated into neighboring aldC-negative bands and vice versa (Figure 5). Mean length of transverse branch was 46.9 ± 3.0 μm (maximum length: 176.8 μm) in GluD2 KO mice, but this value is likely to be an underestimate and actual length would be even larger because these branches were often cut during preparation of histological sections. Therefore, anomalously highly synchronous activation of PCs in the mediolateral axis due to aberrant transverse CF collaterals can spread not only over the boundary of microzones but also beyond the border of zones, indicating that the somatotopic organization of cerebellar cortex of GluD2 KO mice might be disrupted.

### Functional significance

Our findings directly demonstrate the close relationship between anatomical and functional microzonal organization in the cerebellar cortex. Furthermore, it is possible that aberrant transverse wiring affects the firing pattern of IO and DCN neurons through the olivo-cerebellar feedback loop. During postnatal development, only one CF is strengthened, and other surplus CFs are weakened and ultimately eliminated to establish one-to-one relationship between CF and PC (Hashimoto and Kano, 2003, 2005). Results from our on-going reserch suggest that CF-induced calcium transients are highly synchronized among PCs in immature mice and then become desynchronized with development (unpublished data by J.-M. Good, Taisuke Miyazaki, Kenji Sakimura, Masahiko Watanabe, Kazuo Kitamura, Masanobu Kano). Thus, functional differentiation and elimination of redundant CFs during postnatal development are crucial to establish the properly functioning olivo-cerebellar loop.

### Conflict of interest statement

The authors declare that the research was conducted in the absence of any commercial or financial relationships that could be construed as a potential conflict of interest.
